# An Analysis of Drug-Related Problems in the Neurology Ward of a Tertiary Teaching Hospital: A Cross-Sectional Study

**DOI:** 10.7759/cureus.63829

**Published:** 2024-07-04

**Authors:** Milena Borges, João Paulo Vilela Rodrigues, Ana Maria Freato Gonçalves, Marília Silveira Almeida Campos, Fabiana Rossi Varallo, Maria Olivia Barbosa Zanetti, Leonardo Regis Leira Pereira

**Affiliations:** 1 Department of Pharmaceutical Sciences, University of São Paulo, Ribeirão Preto, BRA; 2 Department of Pharmaceutical Sciences, University of São Paulo, Ribeirão Preto, Brazil; 3 Department of Psychiatric Nursing and Human Sciences, University of São Paulo, Ribeirão Preto, BRA

**Keywords:** pharmaceutical research, hospital pharmacy, interested in clinical neurology, pharmaceutical intervention, pharmaceutical care

## Abstract

Background and objective

Drugs that act on the central nervous system have a high potential to cause drug-related problems (DRPs). A clinical pharmacist aided by collaborative efforts within an interdisciplinary healthcare team can prevent, detect, and resolve DRPs, thereby contributing to the promotion of medication safety and improving the quality of life of individuals under care. This study aimed to assess DRPs identified in the neurology ward of a tertiary hospital from February 2016 to November 2019.

Methods

This was a descriptive study with a cross-sectional and retrospective design involving secondary data collected from pharmaceutical care (PC) records. Student's t-tests, Pearson correlation coefficients, Poisson models, and logistic regression models were used to analyze the associations between age, number and type of medications, duration of hospitalization, and the occurrence of DRPs.

Results

A total of 130 patients were included in the study, and a total of 266 DRPs were detected, with 93 patients experiencing more than one DRP and 37 not presenting any DRPs. Necessity-related DRPs were the most prevalent (46.6%) type, followed by safety-related DRPs (28.6%). The prevalence of safety-related DRPs was higher in individuals older than 60 years (p<0.001).

Conclusions

Of note, 84.6% of the interventions suggested by pharmacists to resolve DRPs were accepted by the healthcare team. The high number of DRPs found underscores the importance of the clinical role of the pharmacist and interprofessional collaboration in the care of neurological patients, especially in the pharmaceutical follow-up of elderly individuals.

## Introduction

Neurological health conditions result from neuroanatomical and/or neurophysiological alterations involving the central and/or peripheral nervous system. These neurological diseases are mainly classified into vascular disorders such as stroke, demyelinating diseases, infectious diseases, tumors, hydrocephalus, and cerebral edema, as well as trauma, inflammatory diseases, developmental changes, degenerative diseases, and neuropathies, among others [1]. The clinical manifestations related to such conditions can vary, presenting in either combined or isolated forms, and can occur at different stages of an individual's life and represent a significant cause of morbidity and mortality in the population [1]. However, in Brazil, information regarding hospitalizations in the field of neurology is still limited [2,3].

Studies on morbidity and mortality related to medication use suggest that drugs that act on the central nervous system have a high potential to cause drug-related problems (DRPs) [4]. These are defined as "any undesirable event experienced by the patient that involves or is suspected to involve drug therapy and that actually or potentially interferes with achieving the desired patient outcome."[5] In this context, identifying and evaluating the causes of such DRPs is important for promoting patient safety in the context of neurology [6].

The clinical pharmacist, in the practice of activities related to pharmaceutical care (PC), with the aid of collaborative efforts within the interdisciplinary healthcare team, is capable of preventing, detecting, and resolving DRPs, thus contributing to the promotion of medication safety and improving the quality of life of the individuals under care [5,7]. This study aimed to evaluate the DRPs identified by a clinical pharmacy team in the neurology ward of a tertiary teaching hospital.

## Materials and methods

Study design and population

This was a descriptive study employing a cross-sectional and retrospective design conducted in the neurology ward of the Hospital das Clínicas da Faculdade de Medicina de Ribeirão Preto da Universidade de São Paulo (HCFDRP-USP), a tertiary hospital with teaching, research, and services related to the Brazilian Unified Health System (SUS). The neurology ward caters to adult patients for the investigation or diagnosis of neurological diseases. Patients of both sexes, aged over 18 years at the time of hospital admission, admitted between February 2016 and November 2019, and who were followed up by clinical pharmacists during hospitalization were included. Individuals who remained hospitalized for less than 24 hours were not included. All patients signed the informed consent form before PC follow-up.

Pharmaceutical care

PC was conducted by a clinical pharmacist researcher from Monday to Friday for six hours each day and consisted of medication reconciliation at hospital admission, pharmacotherapeutic follow-up (with a primary focus on daily pharmacotherapy review), and medication reconciliation at hospital discharge. During admission reconciliation, a pharmaceutical anamnesis was conducted through contact with the patient and/or caregiver to obtain sociodemographic data (gender, age, marital status, education, occupation, income) and clinical data (alcohol consumption, smoking, physical activity, dietary habits, allergies, preexisting health conditions, reason for hospitalization, medication use). Information about medications used before admission was compared with the first hospital medical prescription to detect possible discrepancies. If any discrepancy potentially harmful to the patient was detected, a pharmaceutical intervention was performed with the prescribing physician to resolve it. Patient clinical history data were recorded in the medical records using the SOAP (subjective data; objective data; assessment or case study; plan) method.

On each day of hospitalization, for each patient, clinical information was obtained and recorded according to the SOAP [5]. In the assessment ("A"), the identification of real and/or potential DRPs was performed according to the proposed classification: necessity (is there any clinical condition requiring pharmacological therapy that was not proposed? Is there any medication whose use is not justified by the individual's clinical condition? ); adherence (Is the patient adherent to treatment? Could nonadherence be the cause of clinical decompensation and hospitalization? ); effectiveness (Is the indicated pharmacological treatment effective?); and safety (Can any clinical condition presented or patient complaint be explained by the use of medication? Is there a biological plausibility and temporal relationship?) [5]. It is worth noting that adherence is especially important at admission, while the other criteria should be carefully analyzed at admission and during hospitalization.

Medication reconciliation at hospital discharge involved analyzing prescribed and used pharmacotherapy during hospitalization and the patient’s history of medication use. The present study evaluated whether medical prescriptions at discharge were in line with clinical outcomes and with diagnoses or pharmacotherapy adjustments made during hospitalization. Additionally, at discharge, interventions were carried out by the clinical pharmacist to recommend the adequacy of the necessary documentation for medication access via SUS through different components of pharmaceutical assistance, as well as changes in pharmaceutical presentation or medication to another of the same class with similar effectiveness. All these recommendations aimed to ensure more accessible drug therapy, and the DRPs mentioned were classified as adherence-related DRPs since nonadjustment could be related to a potential adherence problem due to a lack of access to medication.

Each PRP or potential PRP detected at each moment of the pharmacist's intervention resulted in at least one interaction with the healthcare team, mainly the physician, through verbal contact and registration in the electronic medical records. All pharmaceutical interventions were also recorded on a specific PC service form (see Appendices). The real or potential DRPs identified by the clinical pharmacist were classified per the following criteria that guided pharmacotherapy review: necessity, adherence, effectiveness, and safety [5]. In addition to PRP classification, the causes of each problem and the acceptance rate of interventions by the healthcare team were described. Elderly individuals were defined as those aged 60 years or older, following the World Health Organization's guidance for developing countries [8]. Medications were identified according to the Brazilian Common Denomination and classified using the ATC - Anatomical Therapeutic Chemical Classification System [9].

Statistical analysis

The database was built in Microsoft Office Excel, and the statistical analyses were performed using the SPSS Statistics version 21.0. (IBM Corp., Armonk, NY). An exploratory analysis of the data was carried out considering measures of central tendency and dispersion. The mean and median were calculated as measures of central tendency, while the standard deviation (SD), interquartile range (IQR), and minimum and maximum values were calculated as measures of dispersion. Qualitative variables were summarized by considering absolute and relative frequencies.

The means of the quantitative variables were compared with those of the sociodemographic variables using Student's t-tests. The Pearson correlation coefficient was used to analyze the correlation between two quantitative variables, and the Poisson model was used to correlate the duration of hospital stay with participants' age. Furthermore, the nature of the relationships between dependent, independent, and confounding variables was explored by adjusting regression models, which are defined according to the nature and distribution of the data. Tests were conducted at a significance level of 5% and a confidence interval of 95%.

This study was performed by adhering to the principles of the Declaration of Helsinki and evaluated and approved by the Research Ethics Committee of the Hospital das Clínicas da Faculdade de Medicina de Ribeirão Preto - USP, under the CAAE (Certificate of Presentation for Ethical Appreciation): 29175414.8.0000.5440. All included patients signed the informed consent form.

## Results

The medical records of 130 patients followed up by pharmacists during the study period were analyzed. The majority of the patients followed were female (n = 75, 57.7%), and (n = 67, 51.5%) were aged under 60 years. These and other sociodemographic data, as well as the cause of hospitalization, are presented in Table 1.

**Table 1:** Sociodemographic data and cause of hospitalization among patients in the study

Variable	N	%
Sex		
Female	75	57.7
Male	55	42.3
Age group		
18-59 years	67	51.5
≥60 years	61	46.9
Data missing	2	1.5
Race		
White	69	53.1
Black	12	9.2
Brown	19	14.6
Data missing	30	23.1
Education		
Up to 8 years	46	35.4
≥8 years	45	34.6
Data missing	27	20.8
Reason for hospitalization		
Diagnostic investigation	69	53.1
Management of previously diagnosed condition	50	38.5
Data missing	11	8.5

The duration of hospital stay was longer among elderly individuals (mean = 16.6 days; SD: 11.1) than among individuals aged 18-59 years (mean = 14.1, SD: 9.5), a result confirmed by the Poisson model (mean difference = 2.31; 95% CI: 0.93; 3.69; Poisson = 10.72; p<0.001). Regarding medication use, individuals aged 60 years or older used, on average, 15.1 medications (SD: 5.6) during hospitalization, while those aged 18 to 59 years used 12.3 medications (SD: 5.7). The analysis by the Poisson model (mean difference = 2.79; 95% CI: 1.5-4.1; Poisson = 17.9; p<0.001) demonstrated the statistical significance of this finding.

Regarding pharmaceutical care, 266 DRPs were identified, with 93 patients (71.53%) presenting one or more DRPs and 37 (28.46%) not presenting any DRPs. The mean number of DRPs per patient was 2.0 (SD: 2.1), ranging from 1 to 10. Analysis of the associations of sex, age, and education with DRPs revealed that elderly patients had a greater risk of developing safety-related DRPs (p<0.001; 95% CI: -0.857; -0.281) (Table 2).

**Table 2:** Association between data variables related to patients in the study ^†^Refers to the number of DRPs found in each category of the variable. ^‡^Student's t-test. ^§^Refers to the Student's t-test EE: elementary education; HE: higher education; CI: confidence interval for the mean difference; DRPs: drug-related problems

Variable	Mean DRPs^†^	± Standard deviation	t^‡^	P-value^§^		95% CI	
Total DRPs							
Male	1.87	2.037	t = -0.379		0.705		-0.875; 0.594
Female	2.01	2.128					
Necessity-related DRPs							
Male	0.95	1.545	t = 0.266		0.791		-0.422;0.553
Female	0.88	1.262					
Effectiveness-related DRPs							
Male	0.51	1.016	t = 0.644		0.521		-0.199;0.390
Female	0.41	0.680					
Safety-related DRPs							
Male	0.53	0.572	t = 3.811		0.700		-0.363;0.245
Female	0.59	1.030					
Total DRPs							
18-59 years	2.01	2.280	t = 0.261		0.795		-0.639; 0.832
60 years or older	1.92	1.882					
Necessity-related DRPs							
18-59 years	1.00	1.537	t = 0.665		0.507		-0.324;0.652
60 years or older	0.84	1.214					
Effectiveness-related DRPs							
18-59 years	0.45	0.909	t = -0.75		0.94		-0.307;0.284
60 years or older	0.46	0.765					
Safety-related DRPs							
18-59 years	0.28	0.572	t = -3.811		<0.001		-0.857;-0.281
60 years or older	0.85	1.030					
Total DRPs							
Illiterate to incomplete EE	2.04	2.160	t = -0.187		0.852		-1.044; 0.865
Complete EE to HE	2.13	2.418					
Necessity-related DRPs							
Illiterate to incomplete EE	1.09	1.363	t = 1.17		0.245		-0.216; 0.834
Complete EE to HE	0.78	1.146					
Effectiveness-related DRPs							
Illiterate to incomplete EE	0.43	0.655	t = -0.544		0.588		-0.459;0.262
Complete EE to HE	0.56	1.036					
Safety-related DRPs							
Illiterate to incomplete EE	0.76	1.079	t = 1.856		0.067		-0.026;0.748
Complete EE to HE	0.40	0.751					

Pearson correlation analysis did not show an association between age (r = 0.044; p = 0.619), length of hospital stay (r = -0.007; p = 0.935), or total number of medications used (r = 0.082; p = 0.354) and the number of DRPs. The most commonly found DRPs were necessity-related (46.6%), especially those related to the lack of indication and medication therapy when indicated based on the scientific literature and clinical protocols (36.5%) (Table 3).

**Table 3:** Classification of DRPs in the study population ^*^DRPs detected at hospital discharge DRPs: drug-related problems; ADR: adverse drug reaction

Type of DRPs	Cause	N (%)
Necessity-related	Untreated condition	97 (36.5)
	Unnecessary treatment	27 (10.2)
Effectiveness-related	Subtherapeutic dosage	29 (10.9)
	Ineffectiveness or potential ineffectiveness	11 (4.1)
	Drug-food interaction	10 (3.8)
	Drug-enteral nutrition interaction	5 (1.9)
	Drug-drug interaction	3 (1.1)
	Drug-enteral tube feeding interaction	2 (0.8)
Safety-related	ADR, ADR potential or contraindication	55 (20.7)
	Overdose	18 (6.8)
	Drug interaction	3 (1.1)
Adherence*	Cost/access	6 (2.3)
Total		266 (100.0)

The medications most involved in DRPs were those that act on the gastrointestinal system and metabolism, with 22.8% corresponding to calcium carbonate or calcium carbonate associated with vitamin D3 and 17.8% to omeprazole. Among medications used to treat the nervous system, antiepileptics accounted for 32.7% of DRPs, with phenytoin accounting for 9.1%. Analysis of the blood and hematopoietic organs revealed that 59.2% of DRPs involved antithrombotic drugs, and 20.4% involved vitamin B12 (Table 4).

**Table 4:** Therapeutic anatomical systems involved in pharmacotherapy-related problems according to the Anatomical Therapeutic Chemical (ATC)* classification ^*^Fonte Anatomical Therapeutic Chemical (ATC): available at https://www.whocc.no/atc_ddd_index/

Systems	N (%)
Gastrointestinal and metabolism	79 (29.7)
Nervous system	55 (20.7)
Blood and hematopoietic organs	49 (18.4)
Cardiovascular system	41 (15.4)
Systemic hormonal drugs, excluding sex hormones and insulin	15 (5.6)
Systemic anti-infectives	13 (4.9)
Antiparasitic	5 (1.9)
Ophthalmological	4 (1.5)
Skin and subcutaneous tissue diseases	2 (0.8)
Musculoskeletal system	2 (0.8)
Respiratory system	1 (0.4)
Total	266 (100.0)

The interventions performed by clinical pharmacists, based on detected DRPs, were accepted by the healthcare team in 84.6% of the situations. Additionally, among the most prescribed medications for the patients included in this study were dipyrone, bromopride, enoxaparin, and omeprazole (see Appendices).

## Discussion

Overall, the incidence of neurological diseases has been on the rise worldwide, especially in elderly patients [10], which warrants conducting studies involving populations affected by neurological conditions. The analysis of our cohort's sociodemographic profiles revealed that the majority of patients were female (n = 75; 57.7%). Autoimmune diseases such as multiple sclerosis and myasthenia gravis are among the most commonly observed conditions in hospitalized patients in neurology wards [11]. The higher prevalence noticed in women is due to the association between the complement of the X sex chromosome and the development of autoimmunity [12].

Regarding age, a small difference was observed between the elderly and nonelderly adult groups. It is important to note that the age range of individuals affected by neurological diseases may vary according to their diagnosis. Some brain tumors and autoimmune diseases are more common in non-elderly adults. For example, multiple sclerosis is rarely diagnosed after the age of 55 [13]. Additionally, males under 40 years of age are often more prone to trauma and neurological injuries (1). On the other hand, cerebrovascular conditions such as stroke and neurodegenerative diseases such as Parkinson's disease and Alzheimer's disease are more prevalent in the elderly population [1,14]. The sociodemographic profile in this study represents a population of patients who required hospitalization, providing insights into pharmaceutical clinical practice in similar settings [11].

Regarding the characteristics of the study participants, the majority (53.1%) were admitted for diagnostic investigation, which is likely related to the hospital's profile as a reference center and research hub [15]. Despite being a teaching hospital, the overall patient care profile is similar to that of other hospitals of advanced care not affiliated with teaching institutions [16]. Elderly individuals used more medications and stayed hospitalized for longer than younger adults. These results indicate a greater number of morbidities requiring pharmacological treatment in the elderly population [17]. Additionally, aging promotes physiological changes related to chronic inflammation and immunosenescence, which can cause disease and delay the favorable evolution of acute conditions, including infectious diseases [18,19]. According to the analysis of the associations by type of DRP, elderly individuals had a greater risk of developing safety-related DRPs (p<0.001). Besides the greater number of medications used, older people have reduced renal mass and clearance and liver capacity to metabolize drugs, raising their susceptibility to adverse drug events [20,21].

As for the analysis of detected DRPs, necessity-related problems were the most prevalent (n = 124; 46.6%). DRPs related to untreated conditions are commonly observed during medication reconciliation at hospital admission when there is a need to include in the hospital prescription a medication that the individual used in an outpatient setting [22]. Additionally, there was a significant percentage of safety-related DRPs (n = 76; 28.6%), followed by effectiveness-related DRPs (22.6%). These findings emphasize the importance of identifying these DRPs to promote patient safety [23] in the hospital setting and the promotion of therapeutic success, especially in situations where there is underdosing [24]. Regarding the DRPs identified, this study noted adherence problems at the time of hospital discharge, highlighting the importance of measures to facilitate access to medications, especially in the context of patients treated by the public health system (SUS), to promote treatment adherence [25].

Regarding the medications most involved in DRPs, those with effects on the gastrointestinal system and metabolism caused problems more frequently (n = 79; 29.7%). The high frequency of calcium carbonate and vitamin D3, which are indicated for the treatment of osteoporosis, reflects the fact that approximately half of the study population was aged 60 years or older. Despite the recommendation for the hospital use of omeprazole for stress ulcer prophylaxis, the indiscriminate use of proton pump inhibitors is a well-known public health problem at different healthcare levels [26]. There is a high demand for antithrombotic drugs, which have pharmacological activity in the blood and hematopoietic system, in the hospital setting due to their indications as prophylactic anticoagulants. In a neurology ward, their use may be even more common due to the hospitalization of patients with motor deficits. Vitamin B12 is frequently used in neurology units since a deficiency of this vitamin can cause neurological conditions such as dementia and neuropathies [27,28].

Furthermore, a systematic review conducted by Souza et al. showed that drugs that act on the central nervous system were the most commonly involved medications in adverse events. The same authors found that age, number of comorbidities, and number of medications were risk factors for the occurrence of these events [25]. Concerning the healthcare team's adherence to pharmacist interventions, a high acceptance rate was observed (84.6%). This finding is consistent with similar studies previously conducted in hospitals, in the context of neurology [11], across various specialties [29], and on the use of antimicrobials [30]. As for the types of interventions made, medication introduction was the most common, since necessity-related DRPs were the most prevalent type.

This study has certain limitations. The cross-sectional design limited our ability to delve into the association of variables. Additionally, studies based on data collected from medical records or follow-up sheets are associated with potential underreporting of information. Finally, pharmaceutical services were provided during the study period from Monday to Friday. In this sense, interventions were not proposed because the pharmacist was not present in the unit full-time.

Reducing harm caused by medications is one of the main goals of the World Health Organization, which, in 2017, published guidelines to reduce the severe adverse events caused by medications by 50% within five years [8]. The pharmacist plays a key role in any initiative aimed at promoting safe medication use, and this study showed the relevance of integrating this professional into the healthcare team. These findings, such as those demonstrating a greater risk of safety problems among elderly people and a high acceptance rate of pharmacist interventions by healthcare teams, particularly by physicians, corroborate the importance of pharmacists and their ability to detect and resolve DRPs. Future studies should evaluate the correlation between the incidence of DRPs and the types of drugs utilized, primarily using a retrospective design to identify the risk factors for developing DRPs in neurological wards.

## Conclusions

The large number of DRPs found in our study highlights the importance of the pharmacist's clinical role and the significance of interprofessional collaboration in the care of neurological patients, especially in the pharmaceutical follow-up of elderly people, given the importance of preventing and resolving DRPs in this setting.

## Figures and Tables

**Figure 1:**
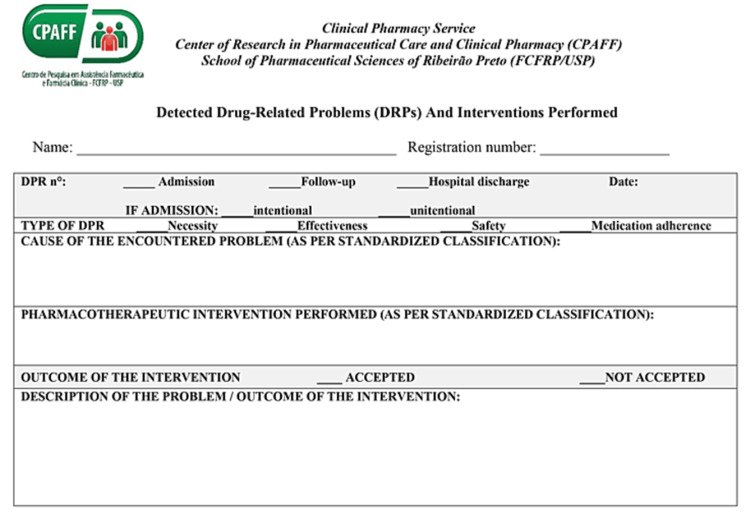
Drug-related problems (DRPs) detected and interventions performed

## Appendices

**Table 5:** Most commonly prescribed medications among patients during hospitalization* *Neurology Ward of HCFMRP-USP. Ribeirão Preto/SP, 2016-2019

Drug	Total, n (%)	Classification ATC	Terapeutic indication²
Dypirone	122 (91.7)	N02BB02	Analgesic and antipyretic
Bromopride	108 (81.2)	AO3FAO4	Gastrointestinal motility stimulant
Enoxaparin	87 (65.4)	B01AB05	Antithrombotic agent
Omeprazole	57 (42.9)	A02BC01	Proton pump inhibitor
Lactulose	46 (34.6)	A06AD11	Laxative
Acetylsalicylic acid	37 (27.8)	N02BA01	Antiplatelet agent
Clonazepam	32 (24.1)	N03AE01	Antiepileptic: benzodiazepine derivative
Losartan	32 (24.1)	C09CA01	Antihypertensive: angiotensin II receptor blocker
Sertraline	31 (23.3)	N06AB06	Antidepressant: selective serotonin reuptake inhibitor
Simvastatin	31 (23.3)	C10AA01	Antilipemic: HMG-CoA reductase inhibitor
Ondansetron	29 (21.8)	A04AA01	Antiemetic and anti-nausea
Hydrochlorothiazide	28 (21.1)	C03AA03	Diuretic
Amlodipine	26 (19.5)	C08CA01	Selective calcium channel blocker
Heparin	26 (19.5)	B01AB01	Antithrombotic agent
Diazepam	25 (18.8)	N05BA01	Benzodiazepine-derived anxiolytic
